# Reduction of Multispecies Biofilms on an Acrylic Denture Base Model by Antimicrobial Photodynamic Therapy Mediated by Natural Photosensitizers

**DOI:** 10.3390/ph17091232

**Published:** 2024-09-18

**Authors:** Ali Shahi Ardakani, Stefano Benedicenti, Luca Solimei, Sima Shahabi, Shima Afrasiabi

**Affiliations:** 1 Laser Research Center of Dentistry, Dentistry Research Institute, Tehran University of Medical Sciences, Tehran 1441987566, Iran; shahi.ali1377@gmail.com; 2 Department of Surgical Sciences and Integrated Diagnostics, University of Genoa, Viale Benedetto XV, 16132 Genoa, Italy; benedicenti@unige.it (S.B.); lucasolimei@hotmail.it (L.S.); 3 Department of Dental Biomaterials, School of Dentistry, Tehran University of Medical Sciences, Tehran 1441987566, Iran

**Keywords:** photochemotherapy, curcumin, riboflavin, phycocyanin, LED, multispecies biofilms, disinfection, natural products

## Abstract

Objectives: The aim of this study is to investigate the antimicrobial efficacy of antimicrobial photodynamic therapy (PDT) using natural photosensitizers (curcumin, riboflavin, and phycocyanin) and light-emitting diode (LED) irradiation against multispecies biofilms in an acrylic denture base model. Materials and Methods: Forty-five acrylic specimens were fabricated using heat-curing acrylic resin. The specimens were then infected with a mixed culture of bacterial and fungal species (including *Streptococcus mutans*, *Streptococcus sanguinis*, *Candida albicans*, and *Candida glabrata*) for 4 days. The acrylic discs were divided into nine groups, with each group containing five discs: control, 0.2% chlorhexidine, 5.25% sodium hypochlorite, curcumin, riboflavin, phycocyanin alone or along with LED. After treatment, the number of colony-forming units (CFUs) per milliliter was counted. In addition, the extent of biofilm degradation was assessed using the crystal violet staining method and scanning electron microscopy. Results: All experimental groups exhibited a significant reduction in colony numbers for both bacterial and fungal species compared to the control (*p* < 0.001). The PDT groups exhibited a statistically significant reduction in colony counts for both bacteria and fungi compared to the photosensitizer-only groups. Conclusions: The results of this in vitro study show that PDT with natural photosensitizers and LED devices can effectively reduce the viability and eradicate the biofilm of microorganisms responsible for causing denture infections.

## 1. Introduction

Partial or total edentulism is a significant health problem that affects various aspects such as aesthetics, speech, chewing ability, dietary habits, and overall quality of life. Treatment usually involves the use of dental prostheses, aiming to restore both the aesthetic appearance and functional requirements of the patient [1,2]. While dental prostheses effectively replace edentulous areas in patients, their long-term use requires careful hygiene and maintenance. Denture stomatitis (DS) can occur as a result of neglecting proper hygiene practices and improper use of these prostheses.

DS is the most prevalent multifactorial chronic inflammatory condition of the oral cavity, typically seen in individuals who wear dentures. This condition affects those who are edentulous and use full or partial dentures, as well as individuals using intraoral removable orthodontic devices and obturators [3]. Between sixty and seventy percent of denture wearers display clinical signs and symptoms of denture stomatitis. If asymptomatic patients are included, this percentage increases to 75% [4,5]. Risk factors associated with denture stomatitis include inadequate denture hygiene, xerostomia, local trauma caused by poorly fitting or inadequately fabricated dentures, prolonged denture use, low salivary pH, smoking, etc. [3,6].

The exact pathogenesis of DS remains unclear; however, infection with *Candida* species is often associated with both local and systemic risk factors [7]. *Candida albicans* is the predominant microorganism associated with denture stomatitis and is commonly found in denture plaque. *C. albicans* has the ability to adhere firmly to irregular and porous denture surfaces, especially those made of acrylic material, resulting in the development of biofilms characterized by a compact extracellular matrix [8,9]. Non-*albicans Candida* species, such as *Candida glabrata*, *Candida krusei*, *Candida tropicalis*, *Candida dubliniensis*, and *Candida parapsilosis* are also known to contribute to the pathogenesis of denture stomatitis [10,11]. It is noteworthy that *C. glabrata* is frequently recognized as the second or third most common cause of infection following *C. albicans*. Moreover, *C. glabrata*’s infections pose a challenge for treatment due to their resistance to numerous antifungal agents [12,13]. Research suggests that bacteria such as *Streptococcus mutans*, *Streptococcus sanguinis*, *Actinomyces odontolyticus*, and *Actinomyces viscosus* can influence the formation of *Candida* biofilms [14]. Compared to individually grown *Candida* cultures, *Candida* in multispecies biofilms exhibit greater invasiveness and persistence [15]. Unfortunately, the number of studies specifically focusing on the inclusion of multiple biofilms is limited [1,16].

In view of the importance of maintaining the hygiene of acrylic dentures by patients, preventing the transmission of infections to medical staff during trial sessions, and avoiding cross-infection in prosthetic laboratories, several methods have been introduced to disinfect these appliances [17]. Acrylic dentures are commonly cleaned by the patient with lukewarm water, a water-vinegar solution, and brushing with a non-abrasive toothpaste. For disinfection, the dentures are immersed in various agents, including glutaraldehyde, isopropyl alcohol, chlorhexidine (CHX), and sodium hypochlorite (NaOCl) [18]. The use of these highly effective chemicals could affect the surface texture and appearance of the denture base [19]. Newer methods, including ultrasonic cleaners and microwaves, have been introduced for the disinfection of acrylic dentures. High-frequency electromagnetic radiation, such as microwaves, has been suggested as a cost-effective and simple method for cleaning dentures, as it effectively induces cell death of microorganisms [20]. Of course, long-term use of this method is not without its drawbacks and may lead to damage to the denture base or even bleaching [21,22]. It should be noted that despite the variety of methods currently available, there is still potential for the development and adoption of new and effective approaches to disinfecting acrylic dentures.

As an alternative to traditional antimicrobial treatments, antimicrobial photodynamic therapy (PDT) is becoming increasingly important due to its ability to effectively combat a wide range of microorganisms. Its use is steadily increasing in various areas of dentistry [23]. PDT is a method in which photosensitizers are activated by irradiation with light of a specific wavelength with oxygen present. This process generates free radicals and reactive oxygen species, which have the potential to induce cell death [24]. Previous research has demonstrated the effectiveness of this approach in combating pathogenic bacteria and fungi found in the oral cavity, with promising results documented [25,26].

The photosensitizers play a pivotal role in determining the efficacy of PDT. The use of natural products is a very effective approach in modern pharmaceutical research and development [27]. This focus on the use of natural products can also be observed in PDT, where efforts are being made to incorporate these substances as photosensitizers [28]. Curcumin, a bioactive phenolic compound derived naturally from the rhizome of the turmeric plant, is garnering significant interest because of its diverse and promising pharmacological properties. Curcumin exhibits a broad absorption peak within the wavelength range of 300–500 nm, which aligns with the emission region of blue light [29]. Riboflavin, also known as vitamin B2, is recognized as a photosensitizer capable of causing oxidative damage to microorganisms exposed to blue light [30]. Riboflavin has demonstrated intrinsic antimicrobial activity against both bacteria and fungi [31]. Riboflavin exhibits peak absorption wavelengths at 270 nm, 336 nm, and 445 nm [32]. Furthermore, phycocyanin, a natural blue pigment, has been explored as a photosensitizer due to its minimal dark toxicity [33]. Phycocyanin exhibits an intense Q-band in the region of 500–730 nm, making it well-suited for use in aPDT experiments [34].

Since it is important to achieve thorough and efficient disinfection of acrylic dentures, this study aims to investigate the antimicrobial efficacy of PDT using natural photosensitizers and light-emitting diode (LED) irradiation against multispecies biofilms in an acrylic denture base model.

## 2. Results

### 2.1. Quantifying Viable Cells

**Figure 1:**
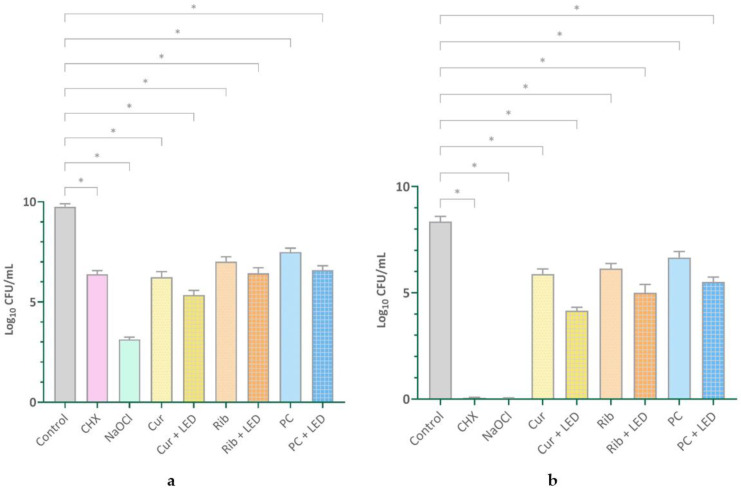
The impact of various experimental groups on the survival of biofilm cells in both bacterial (**a**) and fungal (**b**) species. * Significantly different from the control group, *p* < 0.001. CHX: 0.2% chlorhexidine, NaOCl: 5.25% sodium hypochlorite, Cur: curcumin, LED: light-emitting diode, Rib: riboflavin, PC: phycocyanin.

Figure 1 offers a comprehensive depiction, displaying both the mean values and standard deviations across the different study groups. Significant differences between the groups were validated using ANOVA analysis, with a statistical significance level of *p* < 0.001.

All experimental groups for both bacterial and fungal species exhibited significant reductions in colony numbers relative to the control group (*p* < 0.001). In both cases, a statistically significant reduction in colonies was found in the PDT groups compared to the photosensitizer alone (bacterial: Cur vs. Cur + LED, *p* < 0.001; Rib vs. Rib + LED, *p* = 0.005; PC vs. PC + LED, *p* < 0.001; fungal: photosensitizer vs. photosensitizer + LED, all, *p* < 0.001). The NaOCl group had the lowest colony-forming units (CFUs)/mL count in both bacterial and fungal species, demonstrating a significant difference relative to all other groups (*p* < 0.001). For bacterial species, CHX showed a significant difference compared to all groups except for the groups with LED. For fungal species, excluding the NaOCl group, CHX also demonstrated a significant difference compared to all other groups (*p* < 0.001). 

### 2.2. Biofilm Degradation Assay Results 

**Figure 2:**
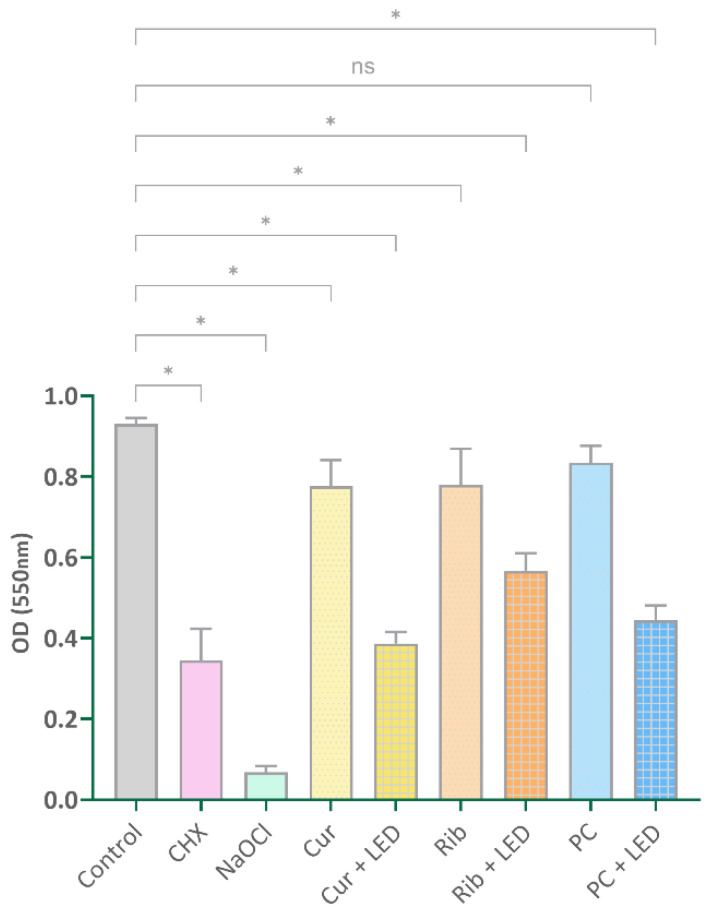
The values corresponding to the optical density (OD) of different groups following crystal violet staining assay. * Significantly different from the control, *p* < 0.001. CHX: chlorhexidine, NaOCl: sodium hypochlorite, Cur: curcumin, LED: light-emitting diode, Rib: riboflavin, PC: phycocyanin.

Figure 2 presents the values associated with the optical density (OD) of various groups following crystal violet staining. 

All experimental groups exhibited significant reductions in OD values compared to the control group (*p* < 0.001), except for the PC group (*p* = 0.119). A statistically significant reduction in OD values was observed in the PDT groups compared to the photosensitizer alone (photosensitizer vs. photosensitizer + LED, *p* < 0.001).

### 2.3. Scanning Electron Microscopy Results 

**Figure 3:**
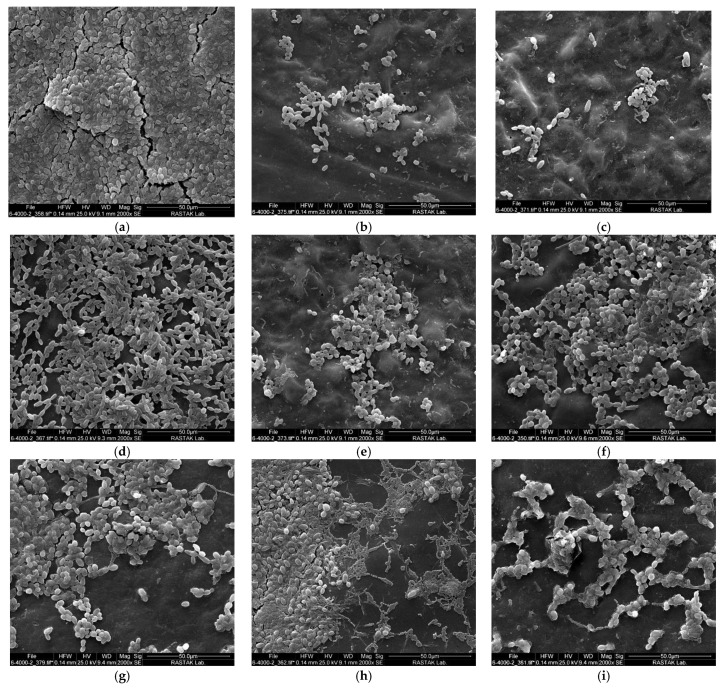
SEM images of different study groups at a magnification of 2000×. (**a**) Control, (**b**) chlorhexidine, (**c**) sodium hypochlorite, (**d**) curcumin, (**e**) curcumin + LED, (**f**) riboflavin, (**g**) riboflavin + LED, (**h**) phycocyanin, and (**i**) phycocyanin + LED.

The scanning electron microscope (SEM) images of the samples after the therapeutic intervention are displayed in Figure 3. As seen in the control group (Figure 3a), a dense biofilm of fungal and bacterial species can be seen, adhering to the surface of the acrylic disc. The CHX and NaOCl groups exhibited remarkable antimicrobial activity, as shown in the images (Figure 3b,c, respectively), where a significant portion of the microbial biofilm was reduced. In the images (Figure 3d,f,h) of the groups in which only dye was used, the biofilm is visible but less dense than in the control group. In the curcumin + LED (Figure 3e), riboflavin + LED (Figure 3g), and phycocyanin + LED (Figure 3i) groups, a notable reduction in biofilm mass was detected after PDT was applied.

## 3. Discussion

Studies have shown that within one year of wearing dentures, there is approximately an eighty percent rise in colonies of *Streptococcus* and *Staphylococcus* species, as well as a thirty percent increase in *C. albicans*, *Klebsiella pneumoniae*, and *Escherichia coli* [35,36]. The growth and development of the *Candida* biofilm is influenced by the presence of multispecies biofilms and other microorganisms. Notably, it has been shown that the extracellular polysaccharide produced by *S. mutans* can increase *C. albicans’* tolerance to antimicrobial agents [2,37,38]. This study used a multispecies biofilm containing *S. mutans*, *S. sanguinis*, *C. albicans*, and *C. glabrata* to contaminate acrylic discs and evaluate antimicrobial activity under more challenging conditions to better simulate the complex environment of the oral cavity.

Given the limitations and drawbacks of current cleaning and disinfection methods for acrylic dentures, ongoing efforts are aimed at finding and introducing a safe and effective alternative [39]. Another key concern connected with the use of antimicrobials is the possible emergence of drug resistance. The intrinsic genetic mechanisms of *C. albicans*, particularly those encoding efflux pumps, are upregulated in response to antifungal treatments. This upregulation facilitates the expulsion of the drugs from the cells and thus plays a crucial role in the development of drug resistance [40,41]. Regarding drug resistance, there are notable differences between the various *Candida* species. For example, *C. glabrata* is more difficult to treat and exhibits greater resistance to common antifungal drugs, such as azoles [42]. Furthermore, *C. albicans*, *C. tropicalis*, and *C. glabrata* cells in biofilms have a significantly higher metabolic activity compared to *C. krusei* [13,43].

The application of PDT represents a remarkable scientific breakthrough in dentistry. It has been thoroughly investigated for its potential in the treatment of microbial infections [44]. PDT offers several advantages over other antimicrobial treatments. Notably, PDT is characterized by a low probability of inducing microbial resistance. Furthermore, PDT is fast-acting and is considered a cost-effective treatment option [45]. It is important to emphasize that the absence of genotoxic and mutagenic effects ensures the long-term safety of PDT [46]. In this study, three natural photosensitizers activated by LED were used to eliminate multispecies biofilm. Based on the findings of this study, the use of these three LED-activated natural dyes resulted in a 3–4 log reduction in the number of microbial colonies compared to the control group (*p* < 0.001). The photosensitizers alone exhibited antimicrobial properties; however, it is noteworthy that their activation with light reduced the number of microorganisms by about 1 to 2 log units. The above results relate to cell viability. Importantly, similar results were observed in the biofilm degradation assay and SEM images. In the absence of light, riboflavin, curcumin, and phycocyanin reduced the biofilm by 16.43%, 16.72%, and 10.25%, respectively. However, the efficacy of these substances in biofilm destruction increased significantly during PDT, reaching 39.08% for riboflavin, 58.48% for curcumin, and 52.19% for phycocyanin.

It is important to note that the number of studies investigating the antimicrobial efficacy of PDT in the acrylic model is limited [16]. A review of previous research on PDT with natural photosensitizers identified only a few in vitro studies for riboflavin and curcumin, while no studies were found for phycocyanin.

Alshehri et al. investigated the efficacy of PDT with 0.1% riboflavin against *C. albicans* biofilm. They employed blue LED light to activate riboflavin, and fungal cell viability was evaluated using the 3-(4,5-dimethylthiazol-2-yl)-2,5-diphenyltetrazolium bromide (MTT) assay. The riboflavin + LED group had the lowest survival rate of *C. albicans*, with less than 50% viability observed. Importantly, the study also evaluated the toxicity of riboflavin alone and riboflavin in combination with LED irradiation against human gingival fibroblast cells, finding a low cytotoxicity profile [47]. This low toxicity is particularly important for acrylic dentures, as they are in constant contact with the oral mucosa throughout the day. In contrast to this study, the above-mentioned study focused only on a biofilm of a single species. In addition, the irradiation duration in their study was longer, lasting 10 min according to the specified irradiation parameters. Other studies have shown the excellent photosensitive properties of riboflavin, which exhibits a high capacity to generate oxygen radical species [48,49]. Its easy availability, lack of side effects and toxicity as well as the ability to inactivate oral pathogens make this substance particularly attractive for clinical applications [50]. 

The antimicrobial properties of curcumin-based PDT have been investigated in various studies [51,52,53]. Quishida et al. examined the efficacy of curcumin-mediated PDT with LED light on biofilms of *C. albicans*, *C. glabrata*, and *S. mutans*. The biofilms were cultivated on acrylic samples for 24 or 48 h and then treated with curcumin and LED light. The results demonstrated that PDT significantly reduced the viability, metabolic activity, and biomass of the biofilms. These findings suggest that curcumin plus LED can effectively disrupt multispecies biofilms, offering potential benefits for dental and medical applications [54]. Regarding the clinical efficacy of curcumin-based PDT, the study by Labban et al. should be emphasized. In this study, the effect of curcumin-mediated PDT was compared with nystatin therapy in the treatment of denture stomatitis in cigarette smokers, a population specifically selected because smoking is a known risk factor for denture stomatitis. The study involved 45 habitual smokers with denture stomatitis. The primary outcome was the number of *Candida* colonies, measured as CFU/mL, with samples collected from the denture surfaces and palatal mucosa at baseline, after 6, and after 12 weeks. The results indicated that *C. albicans* was the most prevalent yeast, followed by *C. tropicalis* and *C. glabrata*. A significant reduction in CFU/mL was observed in the curcumin-mediated PDT group, with clinical efficacy rates of 51% for curcumin-mediated PDT and 49% for nystatin. The study concluded that curcumin-mediated PDT is as effective as nystatin therapy in the treatment of denture stomatitis in smokers [55].

Phycocyanin was employed as a photosensitizer to target microorganisms [56,57,58]. Various studies have demonstrated its efficacy against oral microorganisms, including *Aggregatibacter actinomycetemcomitans* [56], *Enterococcus faecalis* [57], and *S. mutans* and *Lactobacillus acidophilus* dual-species biofilms [58], yielding promising results. This study is the first to use phycocyanin against a mixed biofilm comprising both fungal and bacterial species.

LEDs are commonly used as a light source. They work by applying a voltage to a semiconductor to stimulate charge injection and light emission. The band gap of the semiconductor determines the emission wavelength, which can span from the ultraviolet to the infrared spectrum, depending on the specific material. Many LEDs are inexpensive compared to other light sources and can deliver high irradiances. Although LEDs are not monochromatic or coherent like lamps, their emission spectrum is considerably narrower. Unlike lasers, LEDs are suitable for treating large areas [59,60,61]. Given the need to disinfect acrylic dentures with a large surface area, it is more practical to employ LED devices that can illuminate a larger area. For this reason, LEDs were used in this study. Another important aspect in relation to light is that acrylic resin, as a polymer, is susceptible to degradation via photo-irradiation, mainly through main-chain scission. The threshold wavelength for inducing main-chain scission is <320 nm, which means that irradiation with blue or red LED light does not cause this type of degradation in the polymer [62].

Another important consideration regarding the use of PDT is the impact of this treatment protocol on the mechanical properties of acrylic dentures. In this regard, reference must be made to the recently published systematic review. The review article investigated the effects of PDT on the mechanical properties of denture bases. The study reviewed various research findings to assess the changes in the properties of acrylic resin after PDT (with different photosensitizers, including riboflavin and curcumin), focusing on parameters such as flexural strength, surface hardness, surface roughness, etc. Overall, the report concluded that PDT has no side effects on the mechanical features of acrylic resin and even improves some of its mechanical properties, including wear resistance [1]. However, the efficacy of PDT varies with different photosensitizers, light parameters, and application protocols. Further research is needed to optimize PDT protocols specifically for dental applications and to evaluate the long-term effects on the acrylic material stability and biocompatibility.

Certainly, this study has some limitations. Only heat-cured acrylic resin was used, neglecting other types of acrylics commonly used as denture base. Moreover, the evaluation of antimicrobial efficacy at a single time point cannot adequately demonstrate the full potential of PDT. The results of the study are meaningful under controlled laboratory conditions. However, they may not fully reflect the results in vivo, where oral environmental factors can influence the results. Another important consideration is the potential color change associated with the use of colored materials in PDT. It is crucial to assess their long-term effects on the color stability of acrylic-base dentures, as these photosensitizers may impact the aesthetic appearance of the dentures over time. A notable strength of the study is the use of mixed biofilms containing both fungal and bacterial microorganisms. In addition, the use of a denture base model helps to simulate clinical conditions more realistically. Future studies are recommended to explore various PDT treatment protocols, including multiple applications, as well as to investigate the efficacy of this treatment against resistant microbial strains. Such research could provide comprehensive insights to optimize PDT for clinical use in dentistry.

## 4. Materials and Methods

### 4.1. Fabrication of Acrylic Resin Specimens

Forty-five acrylic specimens, each measuring 12 mm in diameter and 1 mm in thickness, were constructed using heat-cured acrylic resin (ACROPARS 100, Tehran, Iran), a commonly used material in the production of removable dentures. A custom mold of precise dimensions was created using wax and then placed within metal flasks filled with type III dental stone (Pars Dental, Tehran, Iran) for casting. The acrylic resin was prepared using a ratio of 21 g of powder mixed with 10 mL of liquid monomer until it reached a dough-like consistency. Subsequently, the mixture was placed into a cast mold, polymerized, and subjected to bench curing as per standard protocols, involving water immersion at 100 °C for a duration of 30 min. The treated samples were allowed to cool, removed from their flask, and immersed in distilled water at a temperature of 50 °C for a duration of 24 h to eliminate any remaining monomer. The flasks were opened, and the samples were carefully removed and shaped using a metal bur. The specimens were refined using a handheld micromotor (Kavo, Biberach, Germany) equipped with 360, 400, 600, and 1200-grit abrasive papers. Ultimately, the models underwent a polishing process using a wet rag wheel coated with a mixture of coarse pumice and tin oxide. The acrylic discs that had been prepared were subjected to autoclaving at a temperature of 121 °C for a duration of 15 min.

### 4.2. Microorganisms and Biofilm Formation

In this study, the microorganisms, including *S. mutans* (IBRC-M 10682), *S. sanguinis* (ATCC 10556), *C. albicans* (ATCC 10231), and *C. glabrata* (ATCC 90030), were used. The bacterial strains were grown in brain heart infusion (BHI) broth (Merck, Darmstadt, Germany) at a density of 10^8^ CFU/mL. *Candida* strains were cultured in Sabouraud dextrose (SD) broth (Ibresco, Tehran, Iran). Subsequently, individual discs were inoculated with 500 μL of each microbial suspension at a final concentration of 10^7^ CFU/mL for bacterial species and 10^5^ CFU/mL for fungal species in 24-well microplates (SPL Life Sciences, Pocheon, Republic of Korea). The discs were then incubated for 96 h in a 5% CO_2_ environment at 37 °C.

### 4.3. Photosensitizers and Light Sources

Curcumin (Merck, Darmstadt, Germany), riboflavin (BIOCHEM Chemopharma, Cosne-Cours-sur-Loire, France), and phycocyanin (Phoenix, Isfahan, Iran), at a concentration of 10, 10, and 1 mg/mL, respectively, were used as photosensitizers. In this study, a blue LED (Bluedent smart cordless, Plovdiv, Bulgaria) was selected as the light source for the activation of curcumin and riboflavin, as their peak absorption spectra align with the blue wavelength range. The blue LED utilized in the experiment had a wavelength range of 410–490 nm, a power density of 1100 ± 200 mW/cm^2^, an 8 mm beam diameter, a beam area of 0.5 cm^2^, an energy density of 60 J/cm^2^, and operated in continuous mode. To activate phycocyanin, a red LED (FotoSan^®^ 630 LAD pen, Roslev, Denmark) was used because its absorption peak lies within the range of 500 to 730 nm. The red LED used in the experiment had a wavelength range of 630–640 nm, a power density of 2000 ± 400 mW/cm^2^, an 8 mm beam diameter, a beam area of 0.5 cm^2^, an energy density of 60 J/cm^2^, and operated in continuous mode. The duration of exposure to blue and red LED light was 60 s and 20 s, respectively. The light irradiation was performed at a distance of ~1 mm.

### 4.4. Study Groups

Following the completion of the incubation period, the acrylic discs underwent a gentle washing procedure using phosphate-buffered saline (PBS) before being subsequently allocated into nine distinct groups (*n* = 5) in a random manner:

Group 1. Control: The discs were immersed in 1 mL of PBS and maintained under dark conditions for 5 min.

Group 2. CHX: The discs were immersed in 1 mL 0.2% CHX (Hexomex, Tehran, Iran) solution and maintained under dark conditions for 5 min.

Group 3. NaOCl: The discs were immersed in 1 mL 5.25% NaOCl (CobaCid, Cobalt, Tehran, Iran) solution and maintained under dark conditions for 5 min.

Group 4. Curcumin: The discs were immersed in 1 mL curcumin solution and maintained under dark conditions 5 min.

Group 5. Curcumin + blue LED: The discs were immersed in 1 mL curcumin solution and maintained under dark conditions for a duration of 5 min. Then, irradiation was conducted utilizing a blue LED for 60 s per surface of each disc.

Group 6. Riboflavin: The discs were immersed in 1 mL riboflavin solution and maintained under dark conditions for 5 min.

Group 7. Riboflavin + blue LED: The discs were immersed in 1 mL riboflavin solution and maintained under dark conditions for a duration of 5 min. Then, irradiation was conducted utilizing a blue LED for 60 s per surface of each disc.

Group 8. Phycocyanin: The discs were immersed in 1 mL phycocyanin solution and maintained under dark conditions for 5 min.

Group 9. Phycocyanin+ red LED: The discs were immersed in 1 mL phycocyanin solution and maintained under dark conditions for 5 min. Then, irradiation was conducted utilizing a red LED for a duration of 20 s per surface of each disc.

### 4.5. Sampling and Counting of Viable Cells

After treatment, the acrylic discs were rinsed with PBS. The biofilm adhering to each disc was then collected using the vortex for 1 min [63]. Consecutive 10-fold dilutions of the samples were prepared in PBS, followed by plating 10 μL of each dilution onto BHI agar (BioMaxima, Lublin, Poland) supplemented with amphotericin B for quantifying the total cell count of individual microorganisms. Specifically for *C. albicans* and *C. glabrata*, SD agar plates (Ibresco, Tehran, Iran) supplemented with chloramphenicol were used. BHI agar plates were placed in aerobic conditions with 5% CO_2_ and incubated for 24 h at 37 °C, while SD agar plates were incubated aerobically for 24 h at the same temperature. Following the incubation period, the colonies present on the plates were enumerated.

### 4.6. Assessment of Biofilm Degradation by a Colorimetric Assay

Biofilm degradation was assessed using crystal violet (Merck, Darmstadt, Germany) staining method [54]. After treatment, the acrylic discs were rinsed twice with PBS to eliminate unbound cells. Next, 200 μL of 0.1% (*w*/*v*) crystal violet (Merck, Darmstadt, Germany) was added to each disc in 24-well microplates for 15 min and then rinsed with PBS. The bound dye was solubilized with 1 mL of 95% (*v*/*v*) ethanol. Absorbance at 550 nm was measured using a microplate reader (Bio-Tek, Winooski, VT, USA).

### 4.7. Assessment of Biofilm Degradation by a SEM 

After treatment, the acrylic discs were rinsed with PBS at a pH of 7.2. In order to fix the samples, they were immersed in a 2.5% glutaraldehyde solution for 30 min, followed by a 10 min PBS rinse. Post-fixation was conducted using a 1% (wt/vol) osmium tetroxide solution for 20 min, followed by a final PBS rinse. Dehydration was accomplished by exposing the samples to increasing concentrations of ethanol, with each concentration applied for 5 min, starting at 25%, followed by 50%, then 70%, and finally three times at 100%. Following mounting on a substrate, the samples were coated with a layer of gold-palladium. They were examined using a SEM (FEI QUANTA 200 EDAX EDS SILICON DRIFT 2017, Hillsborough, OR, USA) at a magnification of 2000×.

### 4.8. Statistical Analysis

The conformity of CFU/mL data to a normal distribution was confirmed through the Kolmogorov–Smirnov test (*p* < 0.05). Microbial CFU/mL across various groups was compared using one-way ANOVA and Tukey’s test. GraphPad Prism version 10.0.2 (GraphPad Software, Boston, MA, USA) was employed for the analysis, with a significance level established at *p* < 0.05.

## 5. Conclusions

In this study, we used an acrylic denture base model contaminated with various microorganisms, including fungi and bacteria associated with denture stomatitis, to evaluate the antimicrobial efficacy of PDT with natural photosensitizers and LED irradiation. PDT based on natural photosensitizers was found to significantly reduce multispecies biofilms. However, it is important to recognize the limitations of this study, which focused solely on heat-cured acrylic resin and evaluated efficacy at a single time point. Future research should investigate various PDT protocols, including multiple applications and different types of acrylic material, to enhance the clinical relevance and improve the acceptability of PDT in dental practice. Such studies could provide valuable insights for optimizing PDT in practice.

## Data Availability

Data is contained within the article.
